# EGPRN primary care research at a crossroads advancing research on interprofessional collaboration and on integrated, equitable and complex care. An EGPRN Keynote Paper from the Autumn 2025 Plovdiv Conference

**DOI:** 10.1080/13814788.2026.2708494

**Published:** 2026-08-04

**Authors:** Paul Van Royen

**Affiliations:** Faculty of Medicine and Health Sciences, Department of Family Medicine and Population Health, University of Antwerp, Antwerpen, Belgium

**Keywords:** Research agenda, primary care, research methodology, integrated care, interprofessional collaboration

## Abstract

**Background:**

Primary healthcare is facing unprecedented complexity, driven by multimorbidity, social vulnerability, health inequities, rapid digitalisation, and shifting professional roles.

**Recommendations:**

This opinion paper argues that strengthening primary care requires a fundamental reorientation of both research agendas and professional education. We propose a shift towards innovative, theory-informed methodologies capable of capturing how care works in practice, for whom, and under which conditions. Priority research domains include integrated care, interprofessional teamwork, the biopsychosocial health interface, care for vulnerable and superdiverse populations, and the responsible implementation of digital and AI-supported tools.

**Conclusion:**

To capture these dynamics, we advocate for innovative methodologies including longitudinal mixed-methods designs, pragmatic cluster trials, realist evaluation, implementation science, network analysis, and complexity science approaches. Participatory and practice-based research models are essential to ensure relevance and reduce research waste. Finally, we emphasise the need to embed research literacy, interprofessional learning, and digital competence within transformative education for future family physicians.

## KEY MESSAGES

Strengthening primary healthcare requires renewed research agendas and professional education.

Priority domains include integrated and interprofessional care, the mental–physical health interface, vulnerable populations, and digital and AI-supported care.

This requires investment in innovative methodologies such as longitudinal mixed-methods, implementation science and complexity science approaches.

## Introduction

Healthcare systems worldwide are at a critical crossroads, and this transition is particularly pronounced in primary healthcare [1]. Primary healthcare is undergoing profound transformation, driven by escalating clinical and social complexity, rapid technological innovation, and persistent health inequalities, underscoring that access to health and its outcomes remain unevenly distributed.

Contemporary societies are evolving at unprecedented speed, mainly under pressure of five major stress factors: demographic change, climate instability, infectious disease pandemics, economic instability, increasing migration and resulting population superdiversity. Each of these forces amplifies the complexity of health problems presented in primary care, the decision-making processes of clinicians, and the adaptive capacity required from care systems [2].

Within this changing landscape, the role of primary care and family physicians is being fundamentally transformed. Routine and predictable aspects of care are increasingly supported or substituted by telehealth, digital tools, and even patient-led self-management. The clinical core that remains in family physicians’ care is shifting towards more complex, uncertain, multimorbid, psychosocial health needs—domains that require interprofessional collaboration and integrated care [3]. As services continue to transition from hospitals to community-based settings, primary care professionals become key coordinators of this transmural care. Their responsibilities now extend beyond individual patient encounters to include population-level care, patient group management, and attention to lived experience, wellbeing, and social context. Family physicians are expected to act as connectors across multiple health and social care systems, integrating both clinical care and broader dimensions of health and wellbeing.

Much primary care research remains dominated by reductionist designs that insufficiently reflect the dynamic, relational, and context-dependent nature of real-world practice. Successfully navigating this transformation will require leaving the current road and choosing innovative research approaches and a re-orientation of professional training. Future generations of primary care clinicians must be prepared through transformative learning models that reflect the realities of complexity, care integration, environmental sustainability, and digital and AI-augmented practice. At this crossroads, strengthening primary care will depend on advancing research designs and educational strategies that equip future clinicians for expanded professional roles and the multifaceted challenges that accompany them.

*Innovative research* is needed not only to understand how to effectively implement new care models, but also to advance primary care more broadly and address the longstanding and systemic barriers that continue to hinder the realisation of its full potential. To minimise research waste, research questions must be clearly formulated and explicitly grounded in the priorities, contexts, and practical realities of primary care. High-priority research questions can be informed by systematic literature reviews, by an in-depth assessment of daily clinical practice and by knowledge gaps identified in clinical guidelines, where recommendations remain uncertain, contested, or unsupported by robust evidence [4].

This opinion paper is based on the keynote lecture ‘Primary Care at a Crossroads’ at the 101st EGPRN meeting ‘Empowering the next generation of family physicians in a changing healthcare landscape’ on October 16–19, 2025 in Plovdiv, Bulgaria. It discusses four priorities and interconnected domains for future primary care research, in addition to the current research agendas [5,6]. The aim of this paper is to provide a broader approach for a renewed research agenda and innovative research methodologies, as a more adapted answer to the inherent complexity of primary care and integrated care systems.

## Integrated care and interprofessional collaboration

More research *on integrated care and interprofessional collaboration* is needed to build real teamwork across boundaries. Despite decades of projects, we still lack solid evidence on *how* these approaches work best. Existing studies report promising results, yet their transferability remains limited due to high contextual dependency. Conceptual and organisational research from primary care systems in transition may contribute to the evidence base on integrated outpatient care, offering models that align financing, team organisation, and patient benefit, well illustrated by the study on the concept of integrated health care in Bulgaria [7]. Pragmatic real-world trials with a cluster randomised controlled design can better reveal the ‘how’ and ‘why’. Good examples of real-world intervention trials are the study on the effectiveness of care managers for depression treatment [8] or on a proactive diagnostic strategy for early detection of cardiovascular disease [9]. In order to understand implementation *hurdles* and how different professionals, patients, and organisations adapt, these trials should use a multi-perspective approach; perspectives from the patient, the professional and the system are all three important in developing and implementing integrated care and/or interprofessional collaboration. A good example is this cluster randomised controlled trial on the implementation of self-management support for long-term conditions in routine primary care settings [10]. The study results didn’t show any effect of the intervention – but they used a specific theory of implementation for this trial, i.e. the normalisation process theory and they looked at the different perspectives.

## Complexity and vulnerability

Primary care is now the frontline for multimorbidity, mental health and the social determinants of health. Caring for these populations demands *research on complexity and vulnerability.* Research must move beyond disease-based silos to study adaptive care models, holistic approaches, and cultural competence. Especially for multicultural and vulnerable populations, the complexity becomes daily reality. For persons with a migrant background, significant challenges may arise in the context of medical decision-making, where cultural preferences for close family involvement may be difficult to reconcile with the Western model of medicine. The MEDIMIG study is a comprehensive and interdisciplinary research project focused on this complexity in daily practice [11]. Another example of this research agenda domain is the PAR study on prevention and health promotion in a deprived neighbourhood [12] (Box 1).

Box 1. Example of participatory action research.

Van der Vlegel-Brouwer, Eelderink and Bussemaker [12] used a participatory action research (PAR) approach to co-create health promotion and prevention initiatives in a socioeconomically deprived neighbourhood in The Hague, the Netherlands. The study actively involved citizens, healthcare professionals, and community stakeholders in identifying local health priorities, developing interventions, and reflecting on implementation processes. Through iterative cycles of participation, action, and evaluation, the researchers demonstrated how community engagement can strengthen local ownership, enhance trust between stakeholders, and generate contextually relevant solutions to complex health challenges. The study provides a strong example of how participatory methodologies can support integrated, community-oriented primary care and address the social determinants of health in vulnerable populations.

Box 2. Example of mixed-methods design.

Potthoff et al. [24] conducted a mixed-methods feasibility study in Norwegian general practice to explore the implementation of pragmatic case finding for alcohol-related problems during routine consultations. The study combined quantitative data on recruitment, screening uptake, and identification of patients with risky alcohol use with qualitative interviews exploring the experiences of patients and healthcare professionals. The findings demonstrated that opportunistic alcohol case finding can be integrated into everyday primary care practice and was generally acceptable to both clinicians and patients, although practical barriers such as time constraints and workflow integration were identified. By combining effectiveness and implementation outcomes in a real-world setting, the study provides actionable evidence for improving early identification of alcohol-related problems in primary care and illustrates the value of mixed-methods implementation research in family medicine.

## The biopsychosocial health interface

Primary care research should investigate the whole person, not only disease-related aspects – this means exploring and understanding the *health interface between biological, psychological and social dimensions*. We need tools, training, and research designs that reflect this interplay, i.e. longitudinal, person-centred research, grounded in lived experience. Observational survey designs remain valuable for identifying implementation gaps in everyday primary care, such as the underuse of validated mental health screening tools for depressive disorders despite high clinical burden [13]. Any effective intervention should also start from the biopsychosocial health interface and include the different perspectives, especially in the case of psychosocial problems such as for the LGBTQI+ population in primary care [14] or in case of elder abuse. A descriptive qualitative study using face-to-face interviews explored the different stakeholders’ perspectives and the challenging obstacles in preventing, detecting and addressing elder abuse [15]. The findings of this study highlight the complexity of elder abuse, the vulnerability of victims, their reluctance to request help, the potential for abuse of powers of attorney and the tense role of healthcare providers between maintaining a trust-based relationship and the professional obligations of secrecy.

## Digitalisation and artificial intelligence

Artificial intelligence (AI) can support self-management, care transitions, diagnostics and decision-making. The crucial question is how we develop and implement these tools responsibly in primary care. In assessing these tools, we need not only outcomes like accuracy, efficiency and reducing workloads, but also studies on usability, acceptability, sustainability and real-world impact in community care. Co-creative models and participatory design models are essential, bringing developers, patients and primary care professionals together as equal partners throughout development, testing and implementation. AI should not replace the human encounter but rather help us to more focus on it with effective patient communication.

Recently large-language models (LLMs) and generative AI have driven rapid evolution of ambient listening and digital scribing, even within existing clinical workflows. Patient-centred digital scribing may facilitate effective patient communication, for history and physical exam 2.2 to 2.7 times faster than both typing and dictation [16]. Ambient listening leads to major significant increase in usability, reduction in task load and healthcare provider burnout [17]. Most evidence on ambient AI scribes originated outside Europe. This created a critical gap for European primary care, where linguistic diversity, data-governance frameworks (GDPR), and expectations regarding relational continuity of care differ fundamentally. Europe needs its own rigorous, context-sensitive evaluation of usability, acceptability, patient agency, clinical safety, environmental sustainability and ethical impact.

## New research methodologies

Especially in unexplored topics such as social isolation and loneliness among family medicine providers [18] or when contextualised data are missing such as use of validated, culturally-adapted instruments in Eastern Europe primary care practice [19], cross-sectional studies or large-scale surveys can be useful, also to elicit further in-depth research. Also, for specific clinical questions as an answer to knowledge gaps in daily GP practice, randomised clinical trials (RCTs) are very useful. But clinical trials of complex healthcare interventions should be combined with qualitative research. Benefits of integrating qualitative research in clinical trials are well established [20]. Applying mixed methods is useful in assessing the feasibility and implementation of complex interventions [21,22]. Different designs of mixed methods research can be used. To collect different complementary data on the same topic, a convergent parallel design is used, where quantitative and qualitative data collection and analysis are developed simultaneously as equivalent parts, such as in a study on medicine price awareness in chronic patients [23]. In sequential designs, a two-phase process is followed, either to explore a phenomenon by starting with a qualitative research study, such as in the MEDIMIG study [11] or to explain through qualitative research the quantitative data, as in the pragmatic case-finding study to address alcohol use in general practice [24] (Box 2).

In rethinking the research agenda for a new era, we must also move beyond traditional designs such as cross-sectional studies, surveys and RCTs. Primary care research should embrace methodologies that match the complexity of innovative questions, such as realist evaluation trials [25], network analysis [26] and complexity science methodologies [27] (Table 1).

**Table 1.** Overview of innovative research methodologies and examples in primary care research.

Research methodology	Aim/domain	Example (references)
(Longitudinal) mixed method designs	To capture evolving care needs, professional adaptation and patient trajectories, that take time	MEDIMIG project on improving medical decision making in migrant populations [9], study on pragmatic case finding on alcohol use [24]
Pragmatic cluster trials	To evaluate real-world team-based interventions across practices or regions	Study on the effectiveness of care managers for depression treatment [6], study on a proactive diagnostic strategy for early detection of cardiovascular disease [7]
Implementation science frameworks (e.g. Normalisation Process Theory)	To bridge the gap between innovation and practice, and to accelerate uptake of effective innovations	cluster randomised controlled trial on the implementation of self-management support for long term conditions [8]
Realist evaluation and realist-informed implementation trials	To understand what works for whom, and in which contexts or circumstances	Case study of the implementation of advanced nurse practitioner roles in primary care [25]
Network analysis and network science approaches	To study collaboration patterns, knowledge flows, and system interdependencies	Network analysis on interprofessional collaboration in pharmacist-led primary care clinics [26]
Complexity science methodologies	To design multilevel interventions that address the complex needs of individuals receiving care in the context of their lives and communities from a healthcare system that is also complex	Network analysis of multimorbidity and health outcomes among persons with spinal cord injury [27]
Participatory action research	To co-produce research priorities and meaningful outcomes with patients and communities	Participatory action research study on prevention and health promotion in a deprived neighbourhood [12]

MEDIMIG – MEDical decision-making in MIGrant Populations within a super-diverse society.

Practice-based research networks can produce very relevant big data, by using electronic health records, patient registries, health information exchanges, and patient-generated health data. Embedding AI in research is essential as a tool for prediction, personalisation and pattern recognition and AI-assisted data analysis, which will uncover new patterns.

## Transformative learning

Professional education of future primary healthcare providers must keep pace with their changing role in an increasingly more complex context. There is a need for instructional reforms with more interdependence between education and health systems, more competency-based curricula and interprofessional education [28]. Transformative learning is a learning process preparing future healthcare professionals to develop new perspectives, professional identities and leadership, and ways of acting through critical reflection, dialogue and engagement with complex real-world experiences. This also includes a research mindset embedded in the curriculum program and building a peer review community in health professions education [29].

Interprofessional collaboration is a trainable competency, supported by educational research evidence [30]. Studies show that when students learn *with*, *from*, and *about* each other, their understanding of professional roles, shared decision-making and problem-solving improves significantly.

Also, digital competencies should be part of professional competencies. Healthcare providers must learn to integrate digital tools safely and effectively in practice. They should also be able to interact, communicate and collaborate with other professionals and maintain patient-centred care, including when using digital technologies are used.

## Conclusion

To minimise research waste and accelerate scalable innovation, Europe must move from isolated pilot projects to sustained, theory-driven, transnational research programs that generate actionable evidence for integrated, interprofessional, equitable and AI-augmented primary care. There is a need for methodological approaches grounded in complexity science and implementation theory. Preparing the next generation of family physicians requires embedding research literacy, interprofessional training, and digital competence within transformative curricula.

The cost of inaction is system fragmentation, workforce strain, and widening health inequities. The opportunity is a resilient, human-centered, digital and AI-augmented primary healthcare system.
